# Supplementary material for: Discrimination and Characterization of Volatile Flavor Compounds in Fresh Oriental Melon after Forchlorfenuron Application Using Electronic Nose (E-Nose) and Headspace-Gas Chromatography-Ion Mobility Spectrometry (HS-GC-IMS)
Source: Foods. 2023 Mar 16;12(6):1272. doi: 10.3390/foods12061272 (PMC10048207; doi:10.3390/foods12061272)
Supplement: Supplementary file 1 [file foods-12-01272-s001.zip › foods-2118031-supplementary.pdf]

Supporting information

Discrimination and Characterization of Volatile Flavor Compounds in Fresh Oriental Melon after Forchlorfenuron Application Using Electronic Nose (E-Nose) and Headspace-Gas Chromatography-Ion Mobility Spectrometry (HS-GC-IMS)

Qi Wang, Xueying Chen, Chen Zhang, Xiaohui Li, Ning Yue, Hua Shao, Jing Wang and Fen Jin *

Key Laboratory of Agro-Product Quality and Safety, Institute of Quality Standards & Testing Technology for Agro-Products, Chinese Academy of Agricultural Sciences, Beijing 100081, China; wangqi2021yw@163.com (Q.W.); chenxueying0327@163.com (X.C.); lxhui33362@163.com (X.L.)

* Correspondence: jinfen@caas.cn

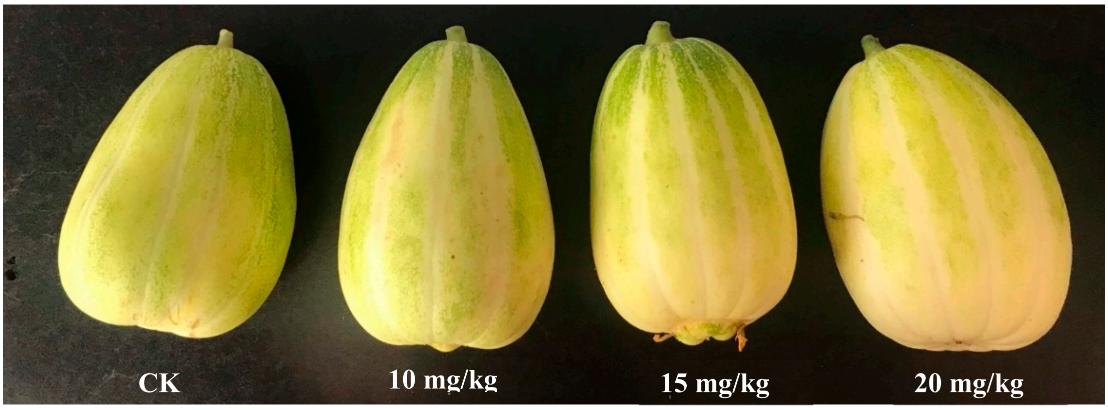

Figure S1. Representative oriental melon fruits at mature period in different application groups.
